# The *mhqPOD* gene cluster in lignin-degrading *Paenibacillus* sp. B2 encodes a pathway for the degradation of lignin-derived 5,5’-di(dehydrovanillic acid) (DDVA)

**DOI:** 10.1093/femsle/fnaf128

**Published:** 2025-11-19

**Authors:** Christos Fanitsios, Robert Millar, Matthew Clegg, Benjamin Dharsi, Robert I Horne, Julia A Fairbairn, Elizabeth M H Wellington, Timothy D H Bugg

**Affiliations:** Department of Chemistry, University of Warwick, Coventry, CV4 7AL, UK; School of Life Sciences, University of Warwick, Coventry, CV4 7AL, UK; Department of Chemistry, University of Warwick, Coventry, CV4 7AL, UK; Department of Chemistry, University of Warwick, Coventry, CV4 7AL, UK; Department of Chemistry, University of Warwick, Coventry, CV4 7AL, UK; Department of Chemistry, University of Warwick, Coventry, CV4 7AL, UK; Department of Chemistry, University of Warwick, Coventry, CV4 7AL, UK; School of Life Sciences, University of Warwick, Coventry, CV4 7AL, UK; Department of Chemistry, University of Warwick, Coventry, CV4 7AL, UK

**Keywords:** Lignin degradation, 5,5’-di(dehydrovanillic acid), DDVA, *Paenibacillus* sp. B2, *Agrobacterium* sp. B1, *Ochrobactrum* sp

## Abstract

Lignin-degrading bacteria *Paenibacillus* sp. B2, *Agrobacterium* sp. B1, and *Ochrobactrum* sp. each contain *mhqO* genes encoding ring cleavage dioxygenase enzymes whose biochemical function is unknown. Each of these strains was found to degrade the biphenyl-containing lignin fragment 5,5’-di(dehydrovanillic acid) (DDVA) on solid media. An operon of five *mhq* genes in *Paenibacillus* sp. B2 was analysed via gene expression using quantitative PCR, and all five genes were highly induced (400–1000-fold overexpression) by the presence of DDVA. Recombinant azoreductase MhqP was found to demethylate DDVA to its monodemethylated derivative. Hence, these genes are proposed to be responsible for DDVA degradation, via a pathway involving the same biochemical steps as that studied in *Sphingobium lignivorans* SYK-6, but using several unrelated genes. Decarboxylation of later pathway intermediate 5-carboxyvanillic acid in *Paenibacillus* sp. B2 is proposed to be catalysed by decarboxylase UbiD, whose gene is also upregulated in the presence of DDVA. Degradation of the other fragment 4-carboxy-2-hydroxypentadienoic acid is proposed to occur via hydratase UxuA, whose gene is also upregulated by DDVA, and 4-hydroxy-4-methyl-2-oxoglutarate aldolase.

## Teaser

The biochemical function of mhq genes in three lignin-degrading bacteria is found to be the degradation of DDVA, a dimeric lignin fragment.

## Introduction

The microbial degradation of lignin, an aromatic heteropolymer found in plant cell walls, is of current interest in biotechnology, since it could enable the conversion of lignin, the most abundant renewable source of aromatic carbon in the biosphere, into high-value chemicals (Schutyser et al. 2018). Lignin is degraded by Basidiomycete fungi, which produce extracellular lignin peroxidase and laccase enzymes to attack the lignin polymer (Martinez et al. 2005, Wong 2009), and by a number of soil bacteria, including *Rhodococcus jostii* RHA1, *Pseudomonas putida* KT2440, and *Streptomyces viridosporus* (Ahmad et al. 2010, Bugg et al. 2021). Although the enzymology of microbial enzymes that can attack lignin is quite well understood (Sodré and Bugg 2024), our understanding of the pathways responsible for the degradation of oxidized lignin fragments is still incomplete (Bugg et al. 2011). It is known that there are key intermediates such as protocatechuic acid and catechol, which are metabolized by the β-ketoadipate pathway (Bugg et al. 2011), which is found in the genomes of the majority of lignin-degrading bacteria (Granja-Travez et al. 2020). Pathways for the degradation of oxidized lignin fragments are well studied in *Sphingobium lignivorans* SYK-6, in which pathways for the degradation of several dimeric lignin fragments have been elucidated (Masai et al. 2007, Takahashi et al. 2014), but it is uncertain whether these same pathways are utilized in other lignin-degrading bacteria. Since such pathways could potentially be used to produce useful bioproducts from lignin degradation via metabolic engineering (Bugg et al. 2021, Sodré and Bugg 2024), there is a need to identify further pathways involved in lignin degradation.

In a survey of aromatic gene clusters present in the genomes of 13 lignin-degrading bacteria published in 2020, we made two observations that led to this present study (Granja-Travez et al. 2020). Firstly, although bacterial lignin degradation usually proceeds via conversion of protocatechuic acid to the citric acid cycle by the β-ketoadipate pathway, two bacteria (*Paenibacillus* sp. B2 and *Lysinibacillus* sp.) lack the β-ketoadipate pathway, so these bacteria must have some alternative pathway that they utilize to degrade lignin fragments. In the genome of *Paenibacillus* sp. B2, the only annotated aromatic ring cleavage dioxygenase genes were *mhqO* and *mhqA* genes, of uncertain biochemical function. Furthermore, *mhqO* genes were also present in the genomes of lignin-degrading *Ochrobactrum* sp. and *Agrobacterium* sp. B1 (Rashid et al. 2017, Granja-Travez et al. 2020). There is genetic evidence that the *mhqO* gene confers resistance to 2-methylhydroquinone in *Bacillus subtilis* (Töwe et al. 2007), and the MhqO protein shares sequence similarity with a ring-cleavage dioxygenase enzyme LinA found in *Sphingomonas paucimobilis* (Miyauchi et al. 1999). There is also a crystal structure of MhqO from *Bacillus subtilis* strain 168 (PDB accession 3OAJ) determined by the New York Structural Genomics Research Consortium, which contains an active site Zn^2+^ metal ion in place of Fe^2+^ which is normally found in catechol dioxygenase enzymes. The MhqA enzyme from *Burkholderia* sp. NF100 has been reported as a flavin-dependent phenol mono-oxygenase enzyme (Tago et al. 2005). However, it is not known whether there is any connection between MhqO and MhqA and bacterial lignin degradation, and what the nature of such a pathway might be. Here, we report a hypothesis that a gene cluster in *Paenibacillus* sp. B2 containing the *mhqO* gene is involved in the degradation of 5,5’-di(dehydrovanillic acid) (DDVA), a biphenyl dicarboxylic acid formed from the microbial degradation of lignin (Chen et al. 1982), whose degradation has been studied previously in *Sphingobium lignivorans* SYK-6 (Peng et al. 1998, Yoshikata et al. 2014), and we report evidence from gene transcription and biochemical studies to support this hypothesis.

## Materials and methods

### Materials

*Paenibacillus* sp. B2, *Agrobacterium* sp. B1, and *Ochrobactrum* sp. were isolated as previously described (Rashid et al. 2017) and were maintained on Luria-Bertani broth or M9 minimal media containing appropriate carbon sources. 5,5’-Di(dehydrovanillic acid) (DDVA) was either synthesized by the method of Elbs and Lerch (1916) or was later purchased from ABCR (UK) Ltd. Hydroxy-DDVA and dihydroxy-DDVA were synthesized via the method of Peng et al. (1998). NMR data: DDVA δ_H_ (300 MHz, d_6_-DMSO) 7.48 (2H, s), 7.44 (2H, s), 3.90 (6H, s, OCH_3_) ppm; hydroxy-DDVA δ_H_ (400 MHz, d_6_-acetone) 7.55 (2H, s), 7.44 (2H, s), 3.81 (3H, s, OCH_3_) ppm; dihydroxy-DDVA δ_H_ (400 MHz, d_6_-DMSO) 9.74 (4H, s, OH), 7.25–7.30 (4H, m) ppm.

### RNA isolation and cDNA synthesis

Total RNA isolation was performed using the Monarch® Total RNA Miniprep Kit according to the manufacturer’s instructions with slight modifications to the sample homogenization procedure. Specifically, *Paenibacillus* sp. B2 was grown in M9 minimal media containing 0.05% DDVA or 0.1% glucose as the growth substrate; *Agrobacterium* sp. B1 was grown in M9 minimal media containing 1% (w/v) Green Value Protobind P1000 soda lignin or 0.2% glucose as the growth substrate. The cell pellet was obtained by harvesting the culture at the mid-exponential phase of growth and centrifuging at 6000 × *g* for 15 min at 4°C and then was resuspended in 50-mM EDTA (450 μl) containing 3-mg/ml lysozyme using vigorous vortexing. cDNA synthesis was performed using the SuperScript™ II Reverse Transcriptase according to the manufacturer’s instructions with the addition of Invitrogen™ Random Primers, and cDNA was stored at −20°C.

### qPCR assay

The 7500 Fast Real-Time PCR System (Applied Biosystem) was used to perform all the qPCR assays. The expression levels of the target genes were normalized relative to stably expressed genes. It was verified that the reference gene had a similar expression level to each tested gene (van der Geize et al. 2001). The normalizing genes chosen for *Paenibacillus* sp. B2 were *rpsU* and *gatB_Yqey* (Reiter et al. 2011); for *Agrobacterium* sp. B1 were citrate synthase, methionyl tRNA ligase, and GAP dehydrogenase.

For each gene, a pair of primers was designed using the BlastP software with a target annealing temperature of 60°C and roughly the same amplicon size of 180–200 bp to allow RT-qPCR reactions to be run in tandem. Each amplicon was used to screen the genome database with the amplicon sequence to ensure that no other are detected in the genome of the strain. Primers used in the present study are listed in Supporting Information Table S2 (for *Paenibacillus* sp. B2) and Table S3 (for *Agrobacterium* sp. B1).

Amplification of the PCR products was performed in a 96-well plate using the Kapa SYBR Fast qPCR Kit Master Mix. The reaction mixture (25 μl) contained 12.5-μl SYBR® Green master mix (Invitrogen), 1-μl primer forward (0.4 μM), 1-μl primer reverse (0.4 μl), 0.6-μl BSA (0.5 mg/ml), and 1-μl DNA template. A nontemplate control for each primer pair was included in all real-time plates to detect any possible contamination. Conditions for qPCR were as follows: 10-min initial denaturation at 95°C, followed by 40 cycles of denaturation for 15 s at 95°C, and 1-min annealing at 60°C. Melt curves were registered at the end of each cycle.

### Enzyme overexpression and purification

The genes for *Paenibacillus* sp. B2 *mhqO* (accession WP_149 645 413) and *mhqP* (accession WP_149 645 414) were codon-optimized for *Escherichia coli* and synthesized (GenScript), then cloned into pET151/D-TOPO expression vector, and transformed into *E. coli* BL21 competent cells (Invitrogen). Cultures of each recombinant strain were grown at 37°C in 1 l of Luria–Bertani media containing 100-μg ml^−1^ ampicillin, induced by the addition of 1-mM IPTG (isopropyl-β-D-thiogalactopyranoside) at OD_600_ = 0.6, and then incubated overnight at 15°C with shaking at 180 rpm. The cell pellet was harvested by centrifugation (6000 *g*, 15 min). The cells were resuspended in 50-mM Tris pH 8.0 containing 10-mM imidazole, 0.5-M NaCl, and 1-mM PMSF, passed through a cell disruptor, centrifuged (10 000 *g*, 35 min), and the supernatant was filtered with a Whatman 0.2-μM syringe filter. The soluble protein fraction was loaded on to a 5-ml pre-equilibrated Ni-NTA column (GE Healthcare) with 20-mM Tris pH 8.0 buffer containing 20-mM imidazole, 0.5-M NaCl, and eluted with 20-mM Tris pH 7.5 containing 300-mM imidazole, 0.5-M NaCl.

### Reaction of MhqP enzyme with DDVA

DDVA (final concentration 1 mM, from 5-mM stock in water adjusted to pH 9.0) was incubated in 50-mM Tris buffer pH 7.5 with NADH (1 mM final concentration) and 50-µg purified MhqP enzyme, total volume 1.0 ml. Control incubations lacking enzyme or NADH, and *Escherichia coli* cell extract, were set up, and samples were incubated at 25°C for 24 h. Aliquots (50 µl) were mixed with methanol (50 µl) and then centrifuged (13 000 rpm, 2 min), and the supernatant was analysed via HPLC. Samples were analysed on a Hewlett Packard Series 1100 analyser, using a Kinetex 5 μm EVO C_18_ reverse phase column (100 Å, 250 × 4.6 mm), with a flow rate of 0.5 ml/min, monitoring at 270 nm. The following gradient was used: 5% MeOH/H_2_O, 0–15 min; 5–10% MeOH/H_2_O, 15–20 min; 10–30% MeOH/H_2_O, 20–25 min; 30–50% MeOH/H_2_O, 25–40 min; 100% MeOH, 40–42 min. Retention times: DDVA, 36.1 min; hydroxy-DDVA, 33.1 min. Control samples containing *E. coli* cell extract showed no conversion of DDVA.

## Results

### Growth on solid minimal media containing DDVA

The growth of several bacteria isolated previously that could degrade polymeric lignin (Rashid et al. 2017) was tested on agar plates containing M9 minimal media supplemented with 0.1% DDVA for 48 h at 30°C (solid media was used due to the low aqueous solubility of DDVA). Strong growth was observed by *Paenibacillus* sp B2, while moderate growth was observed with *Agrobacterium* sp B1 and *Ochrobactrum* sp (see Supporting Information Figure S1). Strong growth on M9/0.1% DDVA was also observed for *Rhodococcus jostii* RHA1, but no growth was observed for *Comamonas testosteroni* or *Lysinibacillus sphaericus* (see Table 1). No growth was observed for *E. coli* K12 on M9/0.1% DDVA. All lignin-degrading bacteria were able to grow on M9 media containing 0.1% vanillic acid as a carbon source (see Table 1).

**Table 1.** Growth of lignin-degrading bacteria on agar plates containing M9 minimal media supplemented with 0.1% DDVA or 0.1% vanillic acid (see Supporting Information Figure S1).

Bacterial strain	Growth on M9/0.1% DDVA^a^	Growth on M9/0.1% vanillic acid^a^
*Paenibacillus* sp. B2	++	++
*Agrobacterium* sp. B1	+	+
*Ochrobactrum* sp.	+	+
*Comamonas testosteroni*	−	+
*Lysinibacillus sphaericus*	−	+
*Rhodococcus jostii* RHA1	++	++

Plates were incubated at 30°C for 48 h.Key: ^a^, ++ strong growth; + moderate growth; − no growth.Isolation of *Paenibacillus* sp. B2, *Agrobacterium* sp. B1, *Ochrobactrum* sp., *Comamonas testosteroni*, and *Lysinibacillus sphaericus* was reported in Rashid et al. (2017).

### Bioinformatic analysis of mhq genes

The genome sequences for *Paenibacillus* sp. B2 (Granja-Travez et al. 2018), *Agrobacterium* sp. B1 (Spence et al. 2020), and *Ochrobactrum* sp. (Granja-Travez et al. 2018) have been previously determined and analysed for genes potentially involved in lignin degradation. There are three annotated *mhqO* genes in the *Agrobacterium* sp. B1 genome (gene ID 195, 660, 1652), and one each in the *Paenibacillus* sp. B2 genome (gene ID 2218) and *Ochrobactrum* sp. genome (gene ID 2571). Pairwise alignments using the Clustal Omega bioinformatics tool (EMBL EBI) revealed that *Agrobacterium* gene 660 aligned poorly with the other sequences, with 15%–17% sequence identity, but that the other four sequences aligned well, with >40% sequence identity (see Supporting Information Figure S2). The active site of the deposited crystal structure PDB 3OAJ contains ligands His-11, His-218, and Glu-266, which are conserved in the sequence alignment.

Adjacent to an *mhqO* gene in each of the three bacteria is a gene annotated as a putative C–C hydrolase, an α/β-hydrolase enzyme which catalyses the C–C bond hydrolysis of extradiol ring cleavage products on aromatic meta-cleavage pathways. Alignment of the three sequences shows > 33% sequence identity in each case, with a conserved GxSxG motif found around the active site serine of this class of enzyme and conserved histidine and aspartate residues that match the positions of the Ser-His-Asp active site residues found in other bacterial C–C hydrolase enzymes (Li et al. 2005) (see Supporting Information Figure S3).

**Figure 1.**
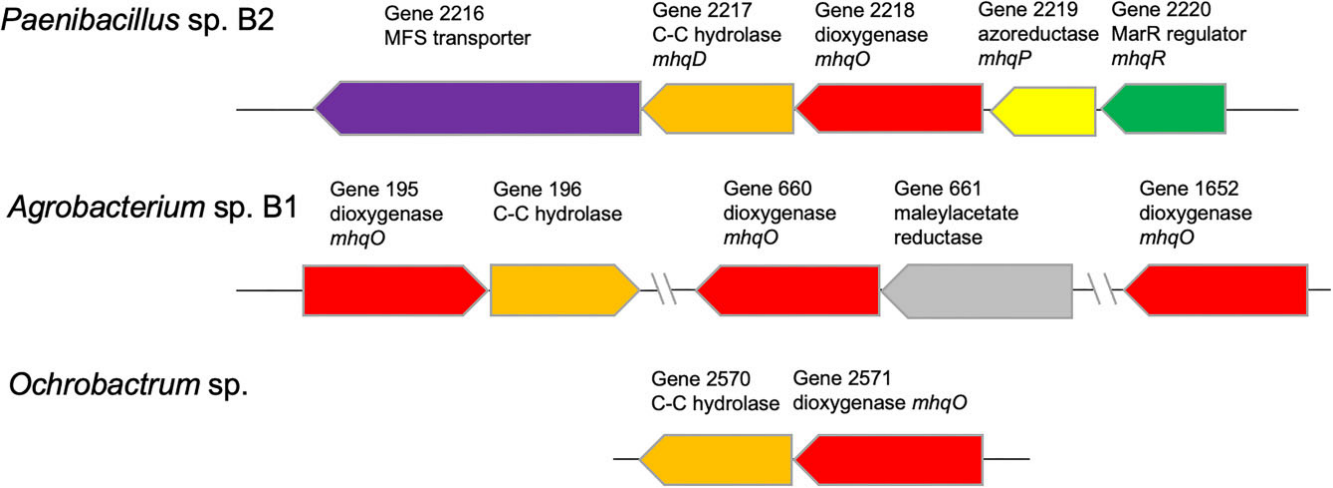
Genomic context of *mhqO* genes in *Paenibacillus* sp. B2, *Agrobacterium* sp. B1, and *Ochrobactrum* sp. Accession numbers are given in Supporting Information (Table S1).

While there is only a single α/β-hydrolase gene adjacent to dioxygenase *mhqO* in the genomes of *Agrobacterium* sp. B1 (genes 195, 196) and *Ochrobactrum* sp. (genes 2570, 2571), in *Paenibacillus* sp. B2 there is a larger operon of five genes (genes 2216–2220), shown in Fig. 1. In addition to the dioxygenase *mhqO* and C–C hydrolase *mhqD*, there is a MarR-type transcriptional regulator (gene 2220), likely to induce the expression of genes in the operon in response to the molecule being degraded, and an MFS transporter (gene 2216), likely to uptake the molecule being degraded. There is also a putative azoreductase MhqP, previously shown in a related gene cluster in *Bacillus subtilis* to confer resistance to 2-methylhydroquinone (Töwe et al. 2007).

**Figure 2.**
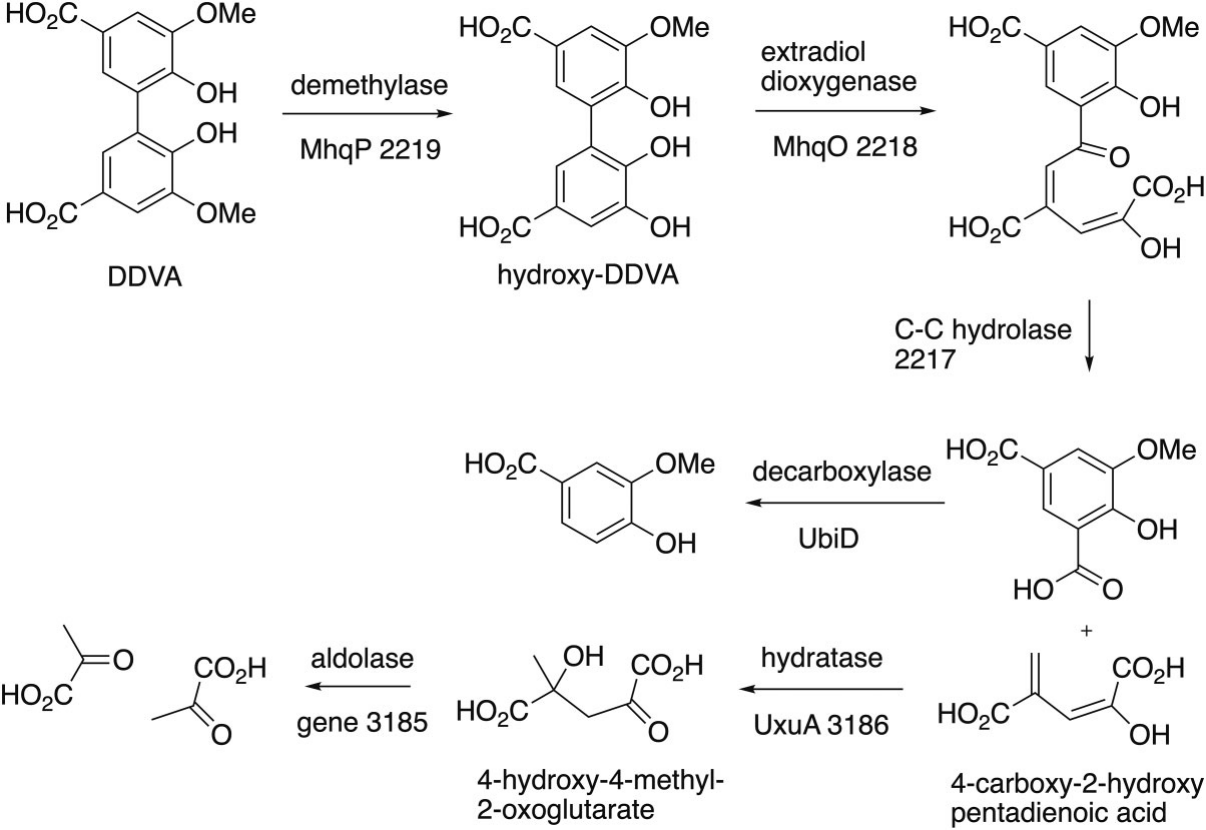
Proposed DDVA degradation pathway for *Paenibacillus* sp. B2, proceeding via extradiol oxidative cleavage, followed by C–C hydrolase bond cleavage. Putative genes responsible in *Paenibacillus* sp. B2 are shown. The later steps of the proposed pathway have not yet been demonstrated biochemically, in particular, the decarboxylation step could occur at an earlier stage.

Since the *mhq* genes are the only genes in the *Paenibacillus* sp. B2 genome annotated as aromatic ring cleavage genes, and since this strain is able to grow on minimal media containing DDVA, we hypothesized that this cluster might be responsible for the degradation of DDVA in these bacteria. The MhqO dioxygenase enzyme clearly appears to be an extradiol catechol dioxygenase, since it contains His, His, Glu motif found in iron(II)-dependent dioxygenases, and the active site of MhqO (PDB 3OAJ) contains a large binding pocket, of sufficient size to accommodate a bicyclic substrate. The MhqD enzyme shares sequence similarity with bacterial C–C hydrolase enzymes found on aromatic meta-cleavage pathways; hence, the presence of these genes implies an extradiol cleavage pathway. Extradiol cleavage of the demethylated hydroxy-DDVA might either be 1,2-oxidative cleavage, as found in the *Sphingobium lignivorans* SYK-6 DDVA degradation pathway (Masai et al. 2007) or 3,4-oxidative cleavage. 1,2-Oxidative cleavage followed by C–C hydrolase cleavage would generate 4-carboxy-2-hydroxy-2,4-pentadienoic acid, which would likely be converted by a hydratase enzyme to generate 4-hydroxy-4-methyl-2-oxoglutarate, followed by aldolase cleavage (see Fig. 2) to generate two equivalents of pyruvate. Bioinformatic searching revealed that each of the three genomes contained a gene encoding 4-hydroxy-4-methyl-2-oxoglutarate aldolase, consistent with such a pathway (see Supporting Information Table S1). Conversely, 3,4-oxidative cleavage would lead to a substituted 2-hydroxymuconate-semialdehyde, which would normally be oxidized by a 2-hydroxymuconate semialdehyde dehydrogenase, but such a gene is not found in the genomes of these bacteria. Furthermore, adjacent to the *Paenibacillus* sp. B2 4-hydroxy-4-methyl-2-oxoglutarate aldolase gene (gene ID 3185) is a hydratase gene *uxuA*, annotated as mannonate dehydratase (gene ID 3186), which potentially might catalyse the hydratase step on the proposed 1,2-cleavage pathway. Therefore, the organization and identity of the genes present in *Paenibacillus* sp. B2 led to the hypothesis that the *mhqROP* gene cluster might encode the initial steps of the pathway shown in Fig. 2.

### Quantitative PCR analysis of gene expression

In order to seek evidence to support this hypothesis, we carried out quantitative PCR analysis of gene expression in *Paenibacillus* sp. B2. Although there was no putative decarboxylase gene related to *Sphingobium lignivorans* SYK-6 *ligW* (Masai et al. 2007), we hypothesized that the decarboxylation step might be catalysed by UbiD/X, shown recently to catalyse decarboxylation of α,β-unsaturated and aromatic carboxylic acids (Marshall et al. 2017, Roberts and Leys 2022); therefore, we also analysed the expression of the *ubiD* gene present in the *Paenibacillus* sp. B2 genome.

**Figure 3.**
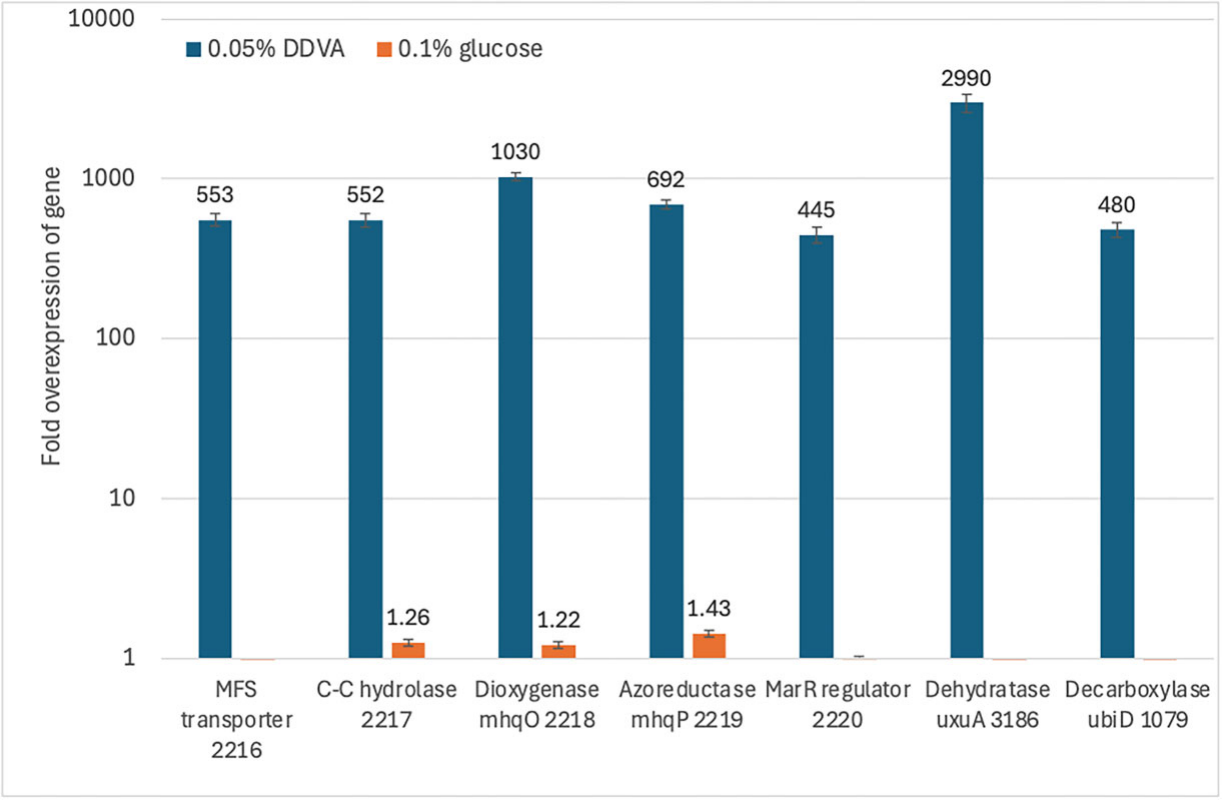
Quantitative PCR analysis of gene expression in *Paenibacillus* sp. B2 in M9 minimal media in the presence of either 0.05% DDVA or 0.1% glucose. Method described in Materials and Methods. Gene expression was normalized relative to that of housekeeping genes *rpsU* and *gatB*.

*Paenibacillus* sp. B2 was grown on M9 minimal media containing 0.05% DDVA, and gene expression was measured in the presence of DDVA and compared with gene expression when grown in the presence of 0.1% glucose. The results, shown in Fig. 3, show high levels of expression of all genes in the cluster in the presence of DDVA, consistent with the induction of the regulatory gene by DDVA and therefore consistent with a role in DDVA degradation. We also observed high levels of overexpression of putative hydratase gene *uxuA*, and decarboxylase *ubiD*, consistent with their involvement in DDVA degradation.

In a separate study, we have also carried out qPCR analysis of genes present in *Agrobacterium* sp. B1 in the presence of 1% Green Value Protobind Lignin P1000 (see Supporting Information Table S4), where we observed a 17-fold overexpression of the *mhqO* gene 1652, with 2.0–2.4-fold overexpression of other *mhqO* genes.

### Biochemical assay of Paenibacillus MhqP

In order to study the enzymes on the pathway, the demethylated hydroxy-DDVA and doubly demethylated dihydroxy-DDVA were synthesized from DDVA using the published synthetic route (Peng et al. 1998). Since the *mhqROP* genes are clustered and are overexpressed in the presence of DDVA, we hypothesized that azoreductase MhqP might catalyse the initial demethylation of DDVA, to form hydroxy-DDVA, and that dioxygenase MhqO then catalyses the oxidative cleavage of hydroxy-DDVA (see Fig. 2).

**Figure 4.**
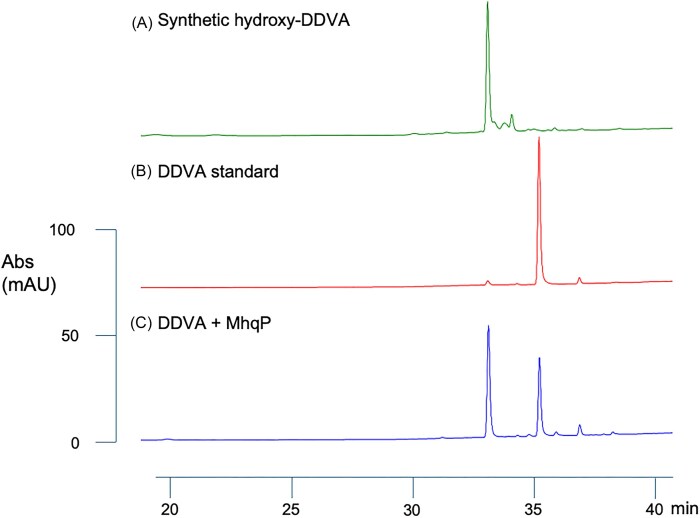
Reverse-phase C_18_ HPLC analysis of enzyme incubation of recombinant MhqP with DDVA. (A) Sample of synthetic hydroxy-DDVA. (B) DDVA standard. (C) Sample of 1-mM DDVA incubated with 50-µg recombinant MhqP for 24 h at 25°C, method described in Materials and Methods. Absorbance recorded at 270 nm. Retention times: DDVA, 36 min; hydroxy-DDVA, 33 min; and dihydroxy-DDVA, 31.5 min.

Recombinant *Paenibacillus* azoreductase MhqP and dioxygenase MhqO were expressed in *E. coli* as His_6_-fusion proteins and purified by Ni-NTA affinity chromatography (see Supporting Information Figure S4). Purified dioxygenase MhqO was unfortunately found to be inactive under a range of assay conditions towards hydroxy-DDVA, dihydroxy-DDVA, protocatechuic acid, or catechol, and no activity could be detected in cell extracts, implying that this dioxygenase enzyme is very unstable *in vitro*, which is sometimes observed for other nonheme iron-dependent dioxygenase enzymes. Azoreductase MhqP was expressed weakly, but the purified enzyme showed activity towards DDVA, as observed by C_18_ reverse phase HPLC (see Fig. 4). DDVA was consumed in the presence of MhqP either in the presence or absence of NADH and was converted into a peak at retention time 33 min matching an authentic sample of hydroxy-DDVA. Hence, there is biochemical evidence to support the role of azoreductase MhqP in catalysing the first step of the proposed pathway.

## Discussion

The hypothesis that the MhqO dioxygenase and associated C–C hydrolase are involved in DDVA degradation is supported by the strong over-expression of *Paenibacillus* sp. B2 *mhqRPOD* genes in the presence of DDVA. Following C–C hydrolase cleavage, we propose that the decarboxylation of 5-carboxyvanillic acid is catalysed by the *Paenibacillus* sp. B2 UbiD, whose gene is upregulated by DDVA. The product of decarboxylation by UbiD is most likely to be vanillic acid, as shown in Fig. 2 but could potentially be iso-vanillic acid (2-hydroxy-3-methoxybenzoic acid), depending on which carboxylic acid is decarboxylated. We suggest that the other product 4-carboxy-2-hydroxypentadienoic acid is degraded via the addition of water by hydratase UxuA (annotated as mannonate dehydratase), which is strongly upregulated by DDVA, and then via C–C cleavage by 4-hydroxy-4-methyl-2-oxoglutarate aldolase. The genes encoding these two enzymes are adjacent on the *Paenibacillus* sp. B2 genome, and there is also a mannonate dehydratase gene (AGRO_5645) situated close to a 4-hydroxy-4-methyl-2-oxoglutarate aldolase gene (AGRO_5642) on the *Agrobacterium* sp. B1 genome (see Supporting Information Table S1). The 4-hydroxy-4-methyl-2-oxoglutarate aldolase genes are annotated on the NCBI database as RraA family proteins; these two protein activities have been shown to be structurally and functionally related by Seah and co-workers (Mazurkewich et al. 2014).

There are similarities and differences between the proposed pathway, and the DDVA degradation pathway of *Sphingobium lignivorans* SYK-6 (Masai et al. 2007), summarized in Table 2. Although the proposed biochemical steps are the same, the initial demethylation step is catalysed in *Sphingobium lignivorans* SYK-6 by a three-component oxidative demethylase (Yoshikata et al. 2014), whereas in *Paenibacillus* sp. B2, this step is catalysed by flavin-dependent azoreductase MhqP. The dioxygenase MhqO shares < 15% amino acid sequence identity with *S. lignivorans* LigZ, and < 10% sequence identity with BphC enzymes on bacterial biphenyl degradation pathways. There is no homologue in *Paenibacillus* sp. B2 for decarboxylase enzyme LigW used for decarboxylation of 5-carboxyvanillic acid in *S. lignivorans* SYK-6 (Masai et al. 2007).

**Table 2.** Comparison of steps in DDVA degradation pathway between *Sphingobium lignivorans* SYK-6 (Masai et al. 2007) and *Paenibacillus* sp. B2.

Biochemical step	*Sphingobium lignivorans* SYK-6	*Paenibacillus* sp. B2	Related?
Demethylation of DDVA	Demethylase LigX	Azoreductase MhqP	No
Extradiol ring cleavage	Dioxygenase LigZ	Dioxygenase MhqO	No
C–C bond hydrolysis	C–C hydrolase LigY	C–C hydrolase MhqD	Yes
Alkene hydration	NI	Mannonate dehydratase UxuA	-
Aldolase bond cleavage	4-Hydroxy-4-methyl-2-oxoglutarate aldolase	4-Hydroxy-4-methyl-2-oxoglutarate aldolase	Yes
Decarboxylation of 5-CVA	Decarboxylase LigW	Decarboxylase UbiD	No

The Table lists the enzymes that catalyse each biochemical step, and whether they are related by sequence similarity. NI, not identified; 5-CVA, 5-carboxyvanillic acid.

The first step of the proposed pathway catalysed by MhqP is demonstrated biochemically. This class of azoreductase enzymes typically breaks down azo dyes (Misal and Gawai 2018) and also has quinone reductase activity (Romero et al. 2020), so demethylation is a new activity for this class of enzyme. The mechanism for flavin-dependent demethylation is not clear, but activity for DDVA conversion was observed either in the presence or absence of NADH (which would be needed to generate reduced flavin), suggesting the involvement of an oxidized flavin cofactor in the reaction mechanism. The formation of a catechol unit in the product might relate to the quinone reductase activity in this class of enzyme (Romero et al. 2020).

We have observed previously that *Paenibacillus* sp. B2 can grow on M9 minimal media containing Kraft lignin (Rashid et al. 2017). Furthermore, another *Paenibacillus* sp. isolate has been reported that can degrade Kraft lignin (Chandra et al. 2008), and a *Paenibacillus glucanolyticus* strain is reported to grow on lignin as carbon source (Mathews et al. 2016). Therefore, perhaps the presence of this DDVA degradation pathway allows this strain to grow on DDVA that is either present in, or generated from, the Kraft lignin growth substrate. In their analysis of the structure of Kraft lignin, Crestini *et al*. (2017) mention a high level of biphenyl units in Kraft lignin, formed by radical couplings in the pulping process. We have previously identified a multicopper oxidase enzyme present in *Paenibacillus* sp. B2 (Granja-Travez et al. 2018), which could potentially release DDVA from oxidative cleavage of biphenyl units present in Kraft lignin.

There are three *mhqO* genes in the genome of *Agrobacterium* sp. B1, one of which (gene 1652) is overexpressed 17-fold in the presence of soda lignin; hence, it seems likely that *mhq* genes are also used in this microbe for the degradation of lignin. There are putative C–C hydrolase, *uxuA* hydratase, and 4-hydroxy-4-methyl-2-oxoglutarate aldolase genes in the genome of this microbe, but the genome of *Agrobacterium* sp. B1 does not contain an *mhqP* gene; therefore, presumably another type of demethylase is present elsewhere in the genome. One of the three *Agrobacterium* MhqO dioxygenases (gene ID 660) shows low sequence similarity with the other MhqO enzymes (see Supporting Information Figure S2), and the genomic context of this gene 660 is different: the neighbouring gene encodes maleylacetate reductase (see Fig. 1), an enzyme which is found on the bacterial hydroxyquinol degradation pathway (Spence et al. 2020). Therefore, it seems probable that this dioxygenase enzyme has a different function from other annotated MhqO enzymes and is perhaps a hydroxyquinol ring cleavage dioxygenase. The genome of *Ochrobactrum* sp. contains a putative C–C hydrolase gene adjacent to its *mhqO* gene and contains a 4-hydroxy-4-methyl-2-oxoglutarate aldolase gene (see Supporting Information Table S1), but there are no annotated *mhqP* or *uxuA* genes present in the genome.

This work establishes a biochemical function for the *mhq* genes found in three lignin-degrading bacteria and indicates that DDVA degradation is carried out by bacteria beyond the Sphingomonad family. The ability to degrade a key lignin fragment is likely to contribute to lignin degradation in soil by microbial consortia.

## Supplementary Material

fnaf128_Supplemental_File
