# Supplementary material for: The mhqPOD gene cluster in lignin-degrading Paenibacillus sp. B2 encodes a pathway for the degradation of lignin-derived 5,5’-di(dehydrovanillic acid) (DDVA)
Source: FEMS Microbiol Lett. 2025 Nov 19;372:fnaf128. doi: 10.1093/femsle/fnaf128 (PMC12673573; doi:10.1093/femsle/fnaf128)
Supplement: fnaf128_Supplemental_File [file fnaf128_supplemental_file.pdf]

The *mhqPOD* genes in lignin-degrading *Paenibacillus* sp. B2 encode a pathway for degradation of lignin-derived 5,5'-di(dehydrovanillic acid) (DDVA)

Christos Fanitsios, Robert Millar, Matthew Clegg, Benjamin Dharsi, Robert I. Horne, Julia A. Fairbairn, Elizabeth M.H. Wellington, Timothy D.H. Bugg*

Supporting Information

Table S1. Accession numbers (NCBI) for genes and enzymes mentioned in this work
Table S2. PCR Primers for qPCR analysis of *Paenibacillus* sp. B2
Table S3. PCR Primers for qPCR analysis of *Agrobacterium* sp. B1
Table S4. qPCR analysis of gene expression in *Agrobacterium* sp. B1 in the presence of 0.1% Green Value Protobind lignin.

Figure S1. Growth of lignin-degrading bacteria on agar plates containing either 0.1% DDVA or 0.1% vanillic acid
Figure S2. Amino acid sequence alignment of dioxygenase MhqO sequences present in *Paenibacillus* sp. B2, *Agrobacterium* sp. B1, and *Ochrobactrum* sp.
Figure S3. Amino acid sequence alignment of C-C hydrolase sequences present in *Paenibacillus* sp. B2, *Agrobacterium* sp. B1, and *Ochrobactrum* sp.
Figure S4. Purification of recombinant *Paenibacillus* sp. B2 MhqO (A) and MhqP (B).

Table S1. Accession numbers (NCBI) for genes and enzymes mentioned in this work

Paenibacillus sp. B2		
Gene	Accession	Annotation
2216	WP_149645411	MFS transporter YdgK
2217	WP_149645412	C-C hydrolase MhqD
2218	WP_149645413	Ring cleavage dioxygenase MhqO
2219	WP_149645414	Oxidoreductase/azoreductase MhqP
2220	WP_081393105	MarR regulator MhqR
3185	KAA1185061	4-hydroxy-4-methyl-2-oxoglutarate aldolase
3186	KAA1185062	Mannonate dehydratase UxuA
1079	KAA1189904	UbiD family decarboxylase
3761	KAA1183323	Mono-oxygenase MhqA
3893	KAA1183444	Mono-oxygenase MhqA
3894	KAA1183445	Flavin reductase
4617	WP_149646908	Mono-oxygenase MhqA
4618	WP_188114662	Flavin mono-oxygenase
4619	WP_149646909	FMN reductase

Agrobacterium sp. B1		
Gene	Accession	Annotation
195	TZG36140	Ring cleavage dioxygenase MhqO
196	TZG36141	C-C hydrolase MhqD
660	TZG36074	Ring cleavage dioxygenase MhqO
661	TZG36073	Maleylacetate reductase
1652	TZG34904	Ring cleavage dioxygenase MhqO
4442	TZG33574	UbiD family decarboxylase
62	WP_149145853	DyP-type peroxidase
1576	WP_149146641	Beta-etherase LigE
3462	TZG34499	4-hydroxy-4-methyl-2-oxoglutarate aldolase
3464	TZG34501	L-arabonate dehydratase
5642	WP_149149148	4-hydroxy-4-methyl-2-oxoglutarate aldolase
5645	WP_149149151	Mannonate dehydratase

Ochrobactrum sp.		
Gene	Accession	Annotation
2570<br>2571	WP_105544626<br>WP_105544627	C-C hydrolase<br>Ring cleavage dioxygenase MhqO
1286<br>1647	PQZ25799<br>PQZ25630	4-hydroxy-4-methyl-2-oxoglutarate aldolase<br>UbiD family decarboxylase

Table S2. PCR Primers for qPCR analysis of *Paenibacillus* sp. B2

Gene target	Primer name	Sequence	Annealing Temperature
2216 MFS transporter	forward	CAGATGGACCCTCACGGAAG	60ºC
	reverse	AGCGACCAACTGACCTGAAG	60ºC
2217 C-C hydrolase MhqD	forward	CAATCTGGTAGCCGTGGGTT	60ºC
	reverse	CCTCAGTCTCCGAAGAAGCG	60ºC
2218 Dioxygenase MhqO	forward	CCTGTCGGTTCTCTGGCTTT	60ºC
	reverse	ACCGAACCCTTTGATCGCAT	60ºC
2219 Azoreductase MhqP	forward	TTTGGATGGTTTGGCGGGTA	60ºC
	reverse	CGCATGCACCTTAACGATGG	60ºC
2220 Regulator MhqR	forward	ACCGGACCCGTATTCCACTA	60ºC
	reverse	CCACATGACGCGGGAAAATC	60ºC
3186 Hydratase uxu	forward	CGATTTGCGAAACAGGCAGG	60ºC
	reverse	TCGCTGCGAATTCAAGTCCT	60ºC
1079 Decarboxylase ubiD	forward	TGCGGTTGGGGAGAAGAAAA	60ºC
	reverse	ATGGGAGTGCTCCCTGTACT	60ºC
rpsU	forward	GTGTCTGAAACGAAAGTTCGCA	60ºC
	reverse	AACTTTCTCTTACGAGCAGCCT	60ºC
gatB_Yqey	forward	TGGACGACAACGAAGTGCT	60ºC
	reverse	GGTTTCCTGGATGGTCTGCT	60ºC

Table S3. PCR Primers for qPCR analysis of *Agrobacterium* sp. B1

Gene	Function	Forward primer	Reverse primer
AGRO_62	Dyp2 peroxidase	CGCATATCACGTCCGCAAAG	ATCGGATCGCTGTCTCCAAC
AGRO_195	MhqO dioxygenase	CTTCACCACGTCACGTCCAT	ATGCGGGAACGGGAAATAGG
AGRO_660	MhqO dioxygenase	CGGCACGAGACGATCAACTA	TGAGAAGTCCGTTTCGGTCG
AGRO_1652	MhqO dioxygenase	CCGTTTCTATACGCCCGAGG	TGGTCGAAGCTGAAATCGGG
AGRO_1576	LigE beta-etherase	GCTGGTGAGCGACAGTTTTG	CGGCTCGTCCAGCATATTGT
AGRO_4442	UbiD decarboxylase	TGACACTCTCGGCAATCAC	ATGATGCGGTAGGAACAGGC
AGRO_1313	Citrate synthase	CAAGGACAAGAACGACCCGT	CGAAGTAGGGGTCGGAAAGG
AGRO_4615	Met-tRNA ligase	AGTTCCATGCGGTCTACTGG	GGCGGAGCAGGTAATAACGA
AGRO_3743	GAP dehydrogenase	CGTACCGACCCCGAATGTTT	GATCGAGGAATGGCTGTCGT

Table S4. qPCR analysis of gene expression in *Agrobacterium* sp. B1. Bacterial cultures were grown in M9 media containing 1% (w/v) Green Value Protobind P1000 soda lignin (50 mL), or 0.2% glucose as a control, at 30 °C, grown to early exponential phase. Gene overexpression in the presence of 1% GVPL is calculated relative to the level of gene expression in the presence of 0.2% glucose. Gene expression was normalised relative to housekeeping genes citrate synthase, methionyl tRNA ligase, and GAP dehydrogenase.

Gene	Accession	Annotation	-fold overexpression in presence of 1% GVPL, relative to expression in 0.2% glucose
195	TZG36140	Dioxygenase MhqO	2.4
660	TZG36074	Dioxygenase MhqO	2.0
1652	TZG34904	Dioxygenase MhqO	17.2
4442	TZG33574	UbiD family decarboxylase	2.7
62	WP_149145853	DyP-type peroxidase	1.8
1576	WP_149146641	Beta-etherase LigE	35

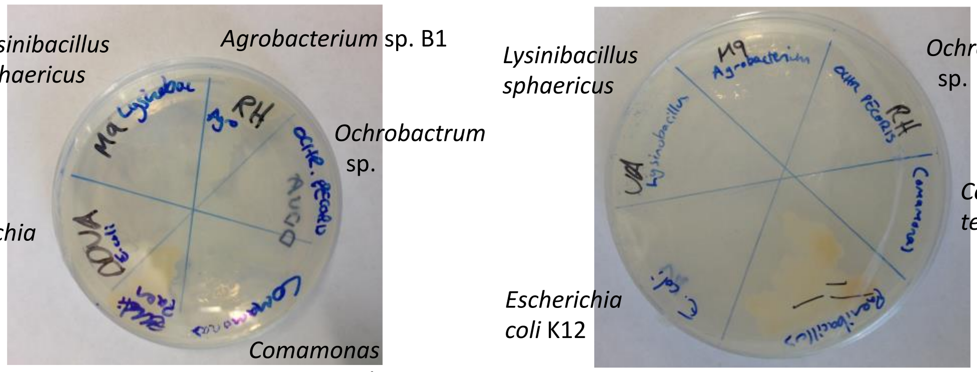

Figure S1. Growth of lignin-degrading bacteria on M9 agar plates containing either 0.1% DDVA (on left) or 0.1% vanillic acid (on right), with no added glucose.

A.

	Paeni 2218	Ochro 2571	Agro 195	Agro 660	Agro 1652
Paenibacillus_2218	-	41	40	16	42
Ochrobactrum_2571	41	-	74	17	66
Agrobacterium_195	40	74	-	15	65
Agrobacterium_660	16	17	15	-	17
Agrobacterium_1652	42	66	65	17	-

B.

```
Paeni2218       MTLQTAGIHHITAFVGDAQRNVDFYAGILGLRLVKKTINFDAPEVYHLYFGNEQGAPGTI      60
Agro_1652       MIKDIKGLHHVTSMASDARQNNSFFTDTLGLRRVKKTVNFDEPSVYHLYYGDEKGTPGTV      60
Ochro2571       MLNQIKGLHHVTSLAADARSNNQFFTDVLGLRRVKKTVNFDEPSVYHLYYGDEVGTPGTV      60
Agro_195        MLNQIKGLHHVTSMAASARQNNNFFTDTLGLRRVKKTVNFDAPDVYHLYYGDEVGTPGSV      60
                *  :  *:**:*::...*: * .*::. **** ****:*** *.*****:*:* *:**::

Paeni2218       ITFFPSPVGRKGRIGAGQVGVTTYAIPVGSLAFWQQRLAAYQISVTL-SNRISETYLSFA      119
Agro_1652       MTYFPFANMMTGRPQVGEVGETQFSIPKGSLGFWKERLIAKGAVGIQADTVFGANRLRFT      120
Ochro2571       MTYFPFPGIQRGRGGTGEVGTTVFAVPQGSLGFWKDRFEQSAVKVESEDEAFGEKRIHFR      120
Agro_195        MTYFPFPHIARGRPGTGEVGTTLFSVPEGSLGYWQDRLSKAGVDGLKADEVFGNKRLHFA      120
                :*:**       ** *.*:** * :::* ***.:*::*:            .  :. . : *

Paeni2218       DYDGLRIELVEREEGPLSKWSFGGVPTGHAIKGFGGAVLYSNNPLQTAETLVHTMGMEHI      179
Agro_1652       GPDGDGLALIETGDDARAAWLAEGISEDNAIRGFTGARFNLHDTAAT-EELLRFMGYDRT      179
Ochro2571       GPDGDSFALVEVKDDPRAPWAHEGIDADHAIRGFHSVSMRLRDEGAT-SELLKYMGYQKL      179
Agro_195        GPDGDGFALVEVRDDNRAQWIGNGVGADQAIHGFQQASLRLRDEGAT-AELLKYMGYQEV      179
                . **  : *:*  :.  : *   *:  .:**:** .. :  .:   *   *:: ** :.

Paeni2218       ADGDGYSRYRTTGDL-GNIIDLKTTP-IPLGAGGTGTVHHIAWRAQDDAEQQEWGLHVQS      237
Agro_1652       EVEGNVTRFAIPGGNGADTIDLAALPKTPFARPGAGSVHHIAFAVENREKQLEVRKALMD      239
Ochro2571       DAKDGTTRLIMPEGNGAGVIDLETLPALGRNQQGAGSVHHVAFAVENREKQLEVRKALMD      239
Agro_195        DRQDGVLRLGIPGGNGADFIDIETMPNISGARLGAGSVHHIAFAVENREKQLEVRKALMD      239
                   ..  *     .  .. **: : *       *:*:***:*: .::  :* *    : .

Paeni2218       HGHHPTPVQDRQYFNAIYFRERGGILFEIATDPPGFARDEAVDALGEKLMLPSWYEPHRA      297
Agro_1652       TGYQVTPVIDRDYFWAIYFRTPGGILFEIATNEPGFDRDEDTAHLGEALKLPNRYEPFRN      299
Ochro2571       TGYHVTPVIDRDYFWAIYFRTPGGVLFEVATNEPGFDRDEDTAHLGEALKLPAQHQHLRN      299
Agro_195        TGYQVTPVIDRDYFWAIYFRTPGGVLFEVATNEPGFDRDEDTAHLGEALKLPTQHAHLRS      299
                 *:: *** **:** *****  **:***:**: *** ***    *** * **   :    *

Paeni2218       AIEENLSPFQIREIEVKQG      316
Agro_1652       QIEANLAPLAA--------      310
Ochro2571       YLEEHLDK-----------      307
Agro_195        VLEEGLEPLGDEKAV----      314
                 :* :*
```

C.

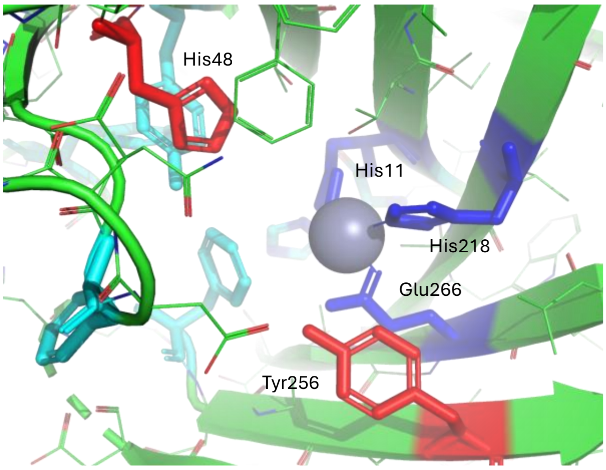

Figure S2. Amino acid sequence alignment of dioxygenase MhqO sequences present in *Paenibacillus* sp. B2, *Agrobacterium* sp. B1, and *Ochrobactrum* sp. A. Pairwise % sequence identity between MhqO sequences; B. Sequence alignment of the four MhqO sequences showing >40% identity, highlighting active site residues; C. Active site of *Bacillus subtilis* MhqO (PDB accession 3OAJ), containing zinc (II) ion, showing metal ligands His-11, His-218 and Glu-266 (highlighted in yellow in sequence alignment), and acid-base residues His-48

and Tyr-256 (highlighted in cyan in sequence alignment). Image generated using Pymol software.

A.

	Paeni 2217	Ochro 2570	Agro 196
Paenibacillus_2217	-	37	33
Ochrobactrum_2570	37	-	62
Agrobacterium_196	33	62	-

B.

```
Paeni2217      --MIHVFH--KGKDVTKPTLVLFHGTGGNEQDLLPLAGLLSPDSSVLGIRGNVLENGMPR     56
Ochro2570      MTIQTYEHRLKAGANGAPLFIVFHGTGGDENQFFGLAEQLLPDATIVSPRGDVSEYGAAR     60
Agro_196       MTRDSYFHKSRAGAAGAPLFVLLHGTGGDENQFFDFGSRLLPEATILSPVGDVSEHGAAR     60
                      *  :.     * ::::*****:*:::: :.  * *:::::.  *:* * *  *

Paeni2217      FFRRLAEGVFDEADLIFRTHEIKQFLDEAAAQYGFDANNLVAVGYSNGANIAGSLLFHYK     116
Ochro2570      FFRRTGEGVYDMEDLARATDKMADFIGGLVAE--HKPSEVIGLGYSNGANIMANLLIEKG     118
Agro_196       FFRRTGEGVYDMADLQRATVKMADFIKANREH--YGAGPVIGLGFSNGANILANVLIEGP     118
               **** .***:*  **   * :: :*:     .  .. . ::.:*:****** ..:*:.

Paeni2217      DVFRTAILLHPMVPLRNITLPSLEGVSIFIGAGTNDPLIASSETEDLEIILQKAGAEVTT     176
Ochro2570      RVFDKAALLHPLVPFRPKDNPVLEGAKILMTAGRMDPICPPDLTEALAHYFERQKAAVEL     178
Agro_196       ELFDAAVLMHPLIPFEPKISPAKASRRVLITAGERDPICPVPLTKALEQSLKAQGGTVET     178
                :*  * *:**::*:.    *   .  ::: **  **:     *: *   ::   . *

Paeni2217      HWGNQGHRLSVAEAEAARDWLQMRSTSTNA  206
Ochro2570      VWHPGGHELRQTELAAVQSLLGK-------  201
Agro_196       VWHPGGHEIRAGEIDAVRGFLATYGE----  204
                *   **.:   *  *.:. *
```

Figure S3. Amino acid sequence alignment of C-C hydrolase sequences present in *Paenibacillus* sp. B2, *Agrobacterium* sp. B1, and *Ochrobactrum* sp. A. Pairwise % sequence identity; B. multiple sequence alignment, highlighting active site residues.

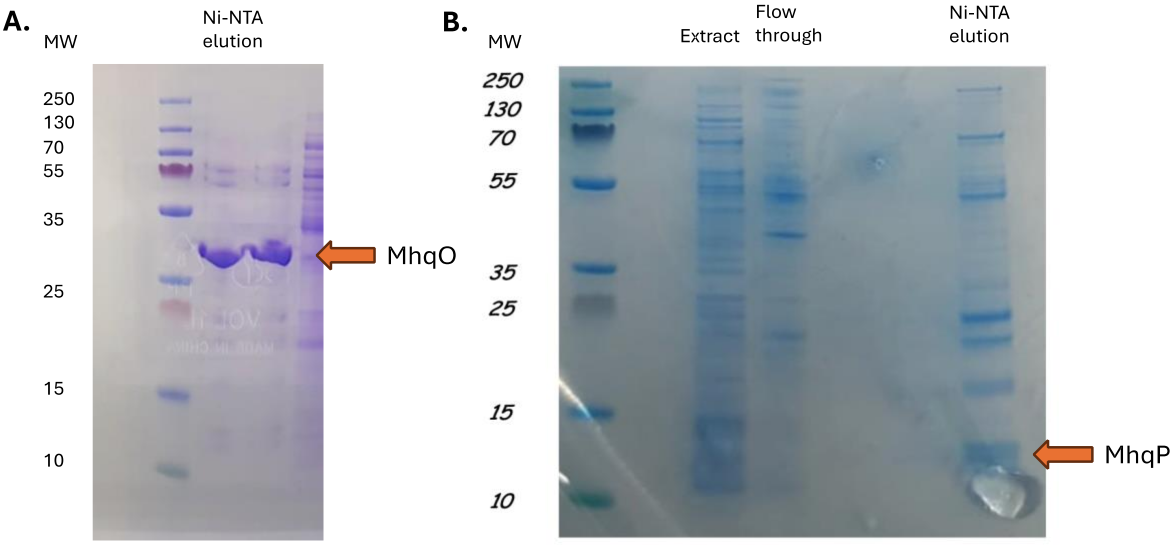

Figure S4. Ni-NTA purification of recombinant *Paenibacillus* sp. B2 MhqO (A) and MhqP (B). Predicted molecular weights: MhqO 34.8 kDa; MhqP 13.5 kDa.
